# ASO Author Reflections: GNAS Mutations and Chemotherapy Response in Appendiceal Adenocarcinoma—Beyond Favorable Histology

**DOI:** 10.1245/s10434-026-19114-1

**Published:** 2026-01-21

**Authors:** Rushabh Gujarathi, Ardaman Shergill

**Affiliations:** 1 Department of Medicine, University of Louisville, Louisville, KY USA; 2 Section of Hematology/Oncology, Department of Medicine, University of Chicago, Chicago, IL USA

## Past

*GNAS-*activating mutations are frequently seen in appendiceal adenocarcinomas (AAs), particularly in the case of mucinous AAs. *GNAS* mutations have been associated with favorable histopathological features and improved overall survival.^1^ However, emerging evidence suggests that *GNAS* mutations are also associated with poor response to systemic therapies commonly used for the treatment of AA.^2^ Hence, we evaluated the predictive utility of *GNAS* mutations for chemotherapy benefit in patients with AA.

## Present

Our study evaluated disease event-free survival (EFS) in 48 patients with metastatic or recurrent AA treated with fluoropyrimidine (5-FU)-based chemotherapy regimens.^3^ Events of interest were death, clinical/radiographic recurrence, or disease progression. Patients with *GNAS*-mutated disease had significantly lower EFS than patients with wild-type tumors (10.7 vs. 20.3 months), even after adjusting for having cytoreductive surgery. Our results suggest that *GNAS* mutations may be independently predictive of decreased therapeutic benefit from 5-FU-based chemotherapies in patients with AAs.

## Future

First, our findings support routine somatic mutation testing for molecular prognostication in patients with AA. Second, we highlight the need for improved therapies in patients with GNAS-mutated AA, given inferior clinical outcomes with standard therapies. Future studies evaluating the role of novel agents such as cyclin-dependent kinase 4/6 inhibitors (e.g., palbociclib) or other targeted agents may warrant further investigation in GNAS-mutated AAs.^4^
